# Chemical composition of cold‐pressed blackberry seed flour extract and its potential health‐beneficial properties

**DOI:** 10.1002/fsn3.1410

**Published:** 2020-01-20

**Authors:** Uyory Choe, Yanfang Li, Lu Yu, Boyan Gao, Thomas T. Y. Wang, Jianghao Sun, Pei Chen, Liangli Yu

**Affiliations:** ^1^ Department of Nutrition and Food Science University of Maryland College Park MD USA; ^2^ Diet, Genomics and Immunology Laboratory Beltsville Human Nutrition Research Center Agricultural Research Service United States Department of Agriculture Beltsville MD USA; ^3^ Food Composition and Methods Development Laboratory Beltsville Human Nutrition Research Center Agricultural Research Service United States Department of Agriculture Beltsville MD USA; ^4^ Institute of Food and Nutraceutical Science School of Agriculture and Biology Shanghai Jiao Tong University Shanghai China; ^5^ Beijing Advanced Innovation Center for Food Nutrition and Human Health Beijing Technology & Business University (BTBU) Beijing China

**Keywords:** anti‐inflammation, antiproliferation, blackberry, ellagitannin, gut microbiota, radical scavenging

## Abstract

The blackberry seed flour was cold‐extracted using 50% acetone and examined for its phytochemical composition and health‐beneficial properties including in vitro gut microbiota modulatory, free radical scavenging, anti‐inflammatory, and antiproliferative capacities. Among identified thirteen components of blackberry seed flour extract through UHPLC‐MS analysis, sanguiin H6 was the primary component and followed by ellagic acid and pedunculagin. For health‐beneficial properties, the blackberry seed flour extract increased the total number of gut bacteria and shifted the abundance of specific bacterial phylum, family, or genus. The extract had RDSC, ORAC, HOSC, and ABTS^•+^ scavenging capacities of 362, 304, 2,531, and 267 μmol Trolox equivalents (TE)/g, respectively. In addition, the blackberry seed flour extract showed capacities for anti‐inflammation and antiproliferation by suppressing LPS induced IL‐1β mRNA expressions in the cultured J774A.1 mouse macrophages and the proliferation of LNCaP prostate cancer cells. The results suggest potential health benefits and further utilization of blackberry seed flour as functional foods.

## Graphical abstract

Blackberry seed flour is a by‐product from oil processing. Investigation of the health‐beneficial components and properties of the blackberry seed flour can lead to its potential utilization in nutraceuticals and functional foods and add value to oil manufacturers and the blackberry producers while reducing environmental contaminations. This study identified the chemical composition of blackberry seed flour using its extract and evaluated potential health‐beneficial properties including in vitro gut microbiota modulatory, free radical scavenging, anti‐inflammatory, and antiproliferative capacities.

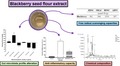

## 1 INTRODUCTION

Blackberries contain many large seeds that are not preferred by consumers. Food industry typically removes seeds when processing blackberries (Bushman et al., 2004). Blackberry seeds are used to produce the seed oil, and the blackberry seed flour is a by‐product from oil processing. Studying the health‐promoting components and properties of the blackberry seed flour can lead to its potential utilization in nutraceuticals and functional foods and create additional value to blackberry producers and related industries while reducing environmental contaminations.

Previously, studies have demonstrated that blackberry seed flour is rich in polyphenolic compounds such as sanguiin H6 and ellagic acid (Hager, Howard, Liyanage, Lay, & Prior, 2008), and has potential free radical scavenging capacity (Bushman et al., 2004). In 2004, Siriwoharn and Wrolstad showed that the 70% acetone (v/v) extracts of both Marion (*Rubus* sp. hyb) and Evergreen (*Rubus laciniatus*) blackberry seeds had significantly greater free radical scavenging capacities compared with whole blackberry or blackberry without seeds (Siriwoharn & Wrolstad, 2004). Also, a few studies have shown anti‐inflammatory capacities of polyphenolic compounds found in blackberry seed flours such as ellagitannins and ellagic acid. It has been reported that these compounds inhibited pro‐inflammatory biomarkers such as IL‐1, IL‐6, and TNF‐α (Landete, 2011). In addition, polyphenolic compounds may play an important role in human health by interacting with gut microbiota (Ozdal et al., 2016). For example, ellagitannins and ellagic acid found in walnut, strawberry, raspberry, and blackberry are metabolized by gut microbiota (Ozdal et al., 2016). Interestingly, a recent study isolated specific bacterial species that is involved in ellagitannins and ellagic acid metabolism (Selma et al., 2017). However, up to date, the influence of blackberry seed flour components on gut microbiota profile has not been evaluated.

In the current study, the chemical composition of blackberry seed flour has been examined. Its potential health‐promoting effects including gut microbiota profile modulatory, free radical scavenging, anti‐inflammatory, and antiproliferative capacities have also been evaluated.

## 2 MATERIALS AND METHODS

### 2.1 Materials and chemicals

Blackberry seed flour was gifted from the Botanic Innovations (Spooner, WI, USA). 2,2′‐Azinobis (2‐amidinopropane) dihydrochloride (AAPH) was purchased from Wako Chemicals (Richmond, VA, USA). Precellys lysing kits were purchased from Bertin Technologies (Rockville, MD, USA). 6‐Hydroxy‐2,5,7,8‐tetramethylchroman‐2‐carboxylic acid (Trolox), 10 ml Pyrex screw‐cap tubes, 37% Hydrochloric acid (HCl), 10 M sodium hydroxide (NaOH), dimethyl sulfoxide (DMSO), and ellagic acid were purchased from Sigma Aldrich (Saint‐Louis, MO, USA). Lysogeny broth (LB) was purchased from Quality Biological™ (Gaithersburg, MD, USA). Iron (III) chloride, fluorescein (FL), 2,2′‐azinobis (3‐ethylbenzothiazoline‐6‐sulfonic acid) diammonium salt (ABTS), DEPC‐treated water and nuclease‐free water, and 30% ACS grade hydrogen peroxide were purchased from Thermo Fisher Scientific (Fair Lawn, NJ, USA). cDNA Synthesis kit was purchased from Agilent Technologies (Savage, MD, USA). J774A.1 and LNCaP cells were purchased from American Type Culture Collection (Manassas, VA, USA). DMEM, RPMI1640, FBS, penicillin, and streptomycin were purchased from GIBCO (Grand Island, NY, USA). QIAamp DNA Mini Kit was purchased from Qiagen (Gaithersburg, MD, USA). TRIzol reagent was purchased from Invitrogen Life Technologies (Carlsbad, CA, USA). TaqMan Fast Universal PCR Master Mix, TATA‐binding protein (TBP) and interleukin‐1beta (IL‐1β) primers, and SYBR®Green Real‐Time PCR Master Mix were purchased from Applied Biosystems (Carlsbad, CA, USA).

### 2.2 Preparation of blackberry seed flour extract

Blackberry seed flour (10 g) was cold‐extracted using 25 ml of 50% acetone at room temperature. Sonication was applied for 1 min, and extraction was performed three times (Choe et al., 2019). The extract's concentration was 0.4 g flour equivalents/mL. All experiments were performed in triplicate.

### 2.3 Ultrahigh‐performance liquid chromatography–high resolution mass spectrometry (UHPLC‐HRMS) analysis

UHPLC‐HRMS analysis was carried out as previously reported (Choe et al., 2019) with an LTQ Orbitrap XL mass spectrometer (Thermo Scientific, Waltham, MA, USA) and Agilent 1,290 infinity liquid chromatography system equipped with a DAD detector. The UV‐vis spectrum scanning range was 190–600 nm. Luna C18 column with 4.6 × 250 mm and 5 μm particle size was used. A formic acid (0.1% v/v) added HPLC grade water was used as solvent A, and a formic acid (0.1% v/v) added acetonitrile was used as solvent B. The elution was 5% of solvent B at the beginning and increased linearly to 13% B at 5 min; increased to 20% B at 10 min; increased to 27% B at 25 min; increased to 33% B at 40 min; increased to 50% B at 45 min; increased to 90% B at 46 min; and keeping 90% B until 51 min; the re‐equilibration postrun time was 10 min. The oven temperature was 40°C, and an injection volume was 5 μL with a flow rate of 1 ml/min. The HRMS was conducted in a negative ionization mode with the optimized parameters as follows: the spray voltage at 4.5 kV, the capillary temperature at 325°C, the capillary voltage at −50 V, and the tube lens offset voltage at −120 V. The mass range was m/z 100–1000 with a resolution of 30,000. Data were postprocessed using the QualBrowser part of the Thermo Scientific Xcalibur 2.2 software. For quantitative analysis, 3.34 ml of blackberry seed flour extract was added to 1.66 ml of 37% HCl in 10 ml Pyrex tube, vortexed for 1 min, and kept at 90°C for 24 hr. The resulted hydrolyte solution was cooled down to ambient temperature, and the pH was adjusted to 2.5 using 5 and 10 M NaOH (García‐Villalba et al., 2015). HPLC grade ellagic acid was used to generate the standard curve and the standard curve was used to quantify the compounds including pedunculagin, sanguiin H6, and ellagic acid derivatives.

### 2.4 Bacterial incubation and microbiota analysis

Gut bacteria were extracted from a chow diet fed C57BL/6J mouse's fecal and cultured in LB following a previously described laboratory protocol (Choe et al., 2019). Bacterial culture and seeding concentration were calculated using wavelength of 600 nm and an OD_600_ value of 1 = 8 × 10^8^ cells/mL. Using 15 ml tubes and 96‐well plates, 1 × 10^7^ cells/mL of bacterial cells were cultured with and without the extract in M9 minimal broth and incubated in a shaker incubator at 37°C for 6 hr. The concentration of flour extract used in the culture medium was 0.4 g flour equivalents/mL. OD_600_ values were measured at 0, 2, 4, 5, and 6 hr. After 6 hr, bacterial cells were centrifuged at 5,000 rpm for 5 min for collection. Precellys lysing and QIAamp DNA mini kits were used to extract the bacterial DNA. Real‐time PCR was performed as reported by a laboratory protocol (Choe et al., 2019). To determine the relative abundance, *Bacteroidetes* and *Firmicutes* phyla, *Enterobacteriaceae* family, *Akkermansia*, *Bifidobacterium*, and *Lactobacillus* genera primers were used.

### 2.5 Relative 2,2‐Diphenyl‐1‐picrylhydrazyl (DPPH) radical scavenging capacity

The DPPH radical scavenging capacity was evaluated according to the laboratory procedure (Cheng, Moore, & Yu, 2006), using a Victor^3^ multilabel plate reader (PerkinElmer, Turku, Finland). DPPH radical solution was prepared in 50% acetone. Trolox was used as the standard. The final reaction mixture contained 0.1 ml of the blackberry seed flour extract, Trolox standard or 50% acetone (the control), and 0.1 ml 0.2 mM DPPH solution. The absorbance was read at 515 nm every minute for 40 min. The relative radical scavenging capacity (RDSC) was calculated from the area under the curve and reported in micromoles of Trolox equivalents/g of dry flour (μmol TE/g).

### 2.6 Hydroxyl radical (HO^•^) scavenging capacity (HOSC)

A fluorescent probe equipped Victor^3^ plate reader (PerkinElmer, Turku, Finland) was used to perform the HOSC assay previously described (Moore, Yin, & Yu, 2006). For a standard, Trolox was used. Fluorescein (170 μL of 9.28 × 10^−8^ M), 30 μL of the solvent, standards, or sample, 60 μL of FeCl_3_, and 40 μL of 0.1990 M of H_2_O_2_ were used as an assay mixture. The concentration of the extract was 40 mg flour equivalents/mL in the reaction mixture. The fluorescence was recorded every 2 min over 4 hr. For excitation and emission, the wavelengths of 485 and 520 nm were used. The HOSC was quantified using the area under the curve and expressed relative to Trolox as μmol TE/g of the dry flour samples.

### 2.7 Oxygen radical absorbing capacity (ORAC)

The oxygen radical absorbing capacity (ORAC) value was determined following a previously reported laboratory procedure (Cheng et al., 2006), with fluorescein (FL) as the fluorescent probe. Trolox standards were prepared in 50% acetone, and other reagents were prepared in 75 mM pH 7.4 phosphate buffer. In the initial reaction, 225 μL of 8.16 × 10^−8^ M FL was combined with 30 μL of sample, standard, or solvent in a 96‐well plate. The plate was heated at 37°C for 20 min in a Victor^3^ multilabel plate reader (PerkinElmer, Turku, Finland). Twenty‐five microliter of 0.36 M AAPH was added to each well, and the fluorescence of the mixture was recorded every 2 min over 2 hr at 37°C. The blackberry seed flour extract's initial concentration was 43 mg flour equivalents/mL in the reaction mixture. For excitation, 485 nm wavelength was used and for emission, 535 nm was used. The ORAC value was reported as μmol TE/g of the dry flour samples.

### 2.8 ABTS^•+^ scavenging capacity

The ABTS radical scavenging capacity of the blackberry seed flour extract was assessed based on a previously reported laboratory protocol (Moore, Cheng, Su, & Yu, 2006). ABTS with manganese oxide was used for ABTS^•+^ working solution preparation. The prepared ABTS^•+^ working solution's absorbance was measured at 734 nm and adjusted to 0.700 ± 0.005. For a standard, Trolox was used. The reaction was carried with 1 ml ABTS^•+^ working solution and 80 μL of the blackberry seed flour extract or standard or solvent. The initial concentration of blackberry seed flour extract was 30 mg flour equivalents/mL in the reaction mixture. The reaction mixture was vortexed for 30 s, and absorbance value was recorded at 734 nm after 90 s of the reaction. The ABTS value was reported as μmol TE/g of the dry flour samples.

### 2.9 Anti‐inflammatory capacity

Anti‐inflammatory capacity was examined based on the laboratory procedure (Whent, Slavin, Kenworthy, & Yu, 2009). The density of 6 × 10^5^ cells/mL J774A.1 mouse macrophages was cultured to reach 80% confluency in DMEM with 10% FBS and 1% penicillin and streptomycin at 37°C under 5% CO_2_ in six‐well plates. Then, macrophages were incubated with the blackberry seed flour extract concentration of 0.4 mg flour equivalents/mL for 48 hr. In every 24 hr, the medium was changed. For inflammatory response, 10 ng/ml lipopolysaccharide (LPS) was delivered to the medium and incubated for 4 hr. After 4 hr, RNA was isolated from the macrophages and cDNA was synthesized using the cDNA synthesis kit. Real‐time PCR was performed using ABI Prism 7,000 Sequence Detection System using TaqMan Universal PCR Master Mix. For primers, TBP was used as a control and an IL‐1β as an inflammatory response. The PCR amplification parameters are as follow: 46 amplification cycles, 50°C for 2 min, 95°C for 10 min, 95°C for 15 s, and 60°C for 1 min (Whent et al., 2009).

### 2.10 Antiproliferative capacity

The antiproliferative capacity was evaluated using the earlier laboratory procedure (Whent et al., 2009). The density of 1 × 10^4^ cells/mL LNCaP prostate cancer cells was cultured with blackberry seed flour extract (0.4 mg flour equivalents/mL). In response to the blackberry seed flour extract delivery, the extract was dissolved in DMSO (1:10 v/v). In a final mixture, the vehicle, 1% DMSO, was delivered for the treatment. For cell proliferation calculation, An ATP‐Lite 1 step kit (Perkin–Elmer Life and Analytical Sciences, Shelton, CT) was used. The Victor^3^ plate reader (Perkin–Elmer, Turku, Finland) was used to determine the emitted luminescence. Media were changed daily, and data were collected at five different time points including 0, 24, 48, 72, and 96 hr. All experiments were performed in triplicate.

### 2.11 Statistics

For statistical analysis, PRISM8 software from GraphPad was used. Each data point was obtained through means ± standard deviation (*SD*). For the value comparison, multiple *t* tests or a one‐way analysis of variance (ANOVA) (*p* ≤ .05) followed by a post hoc test (Tukey's test) was used.

## 3 RESULTS AND DISCUSSION

### 3.1 Chemical composition of the blackberry seed flour extract

**Figure 1:**
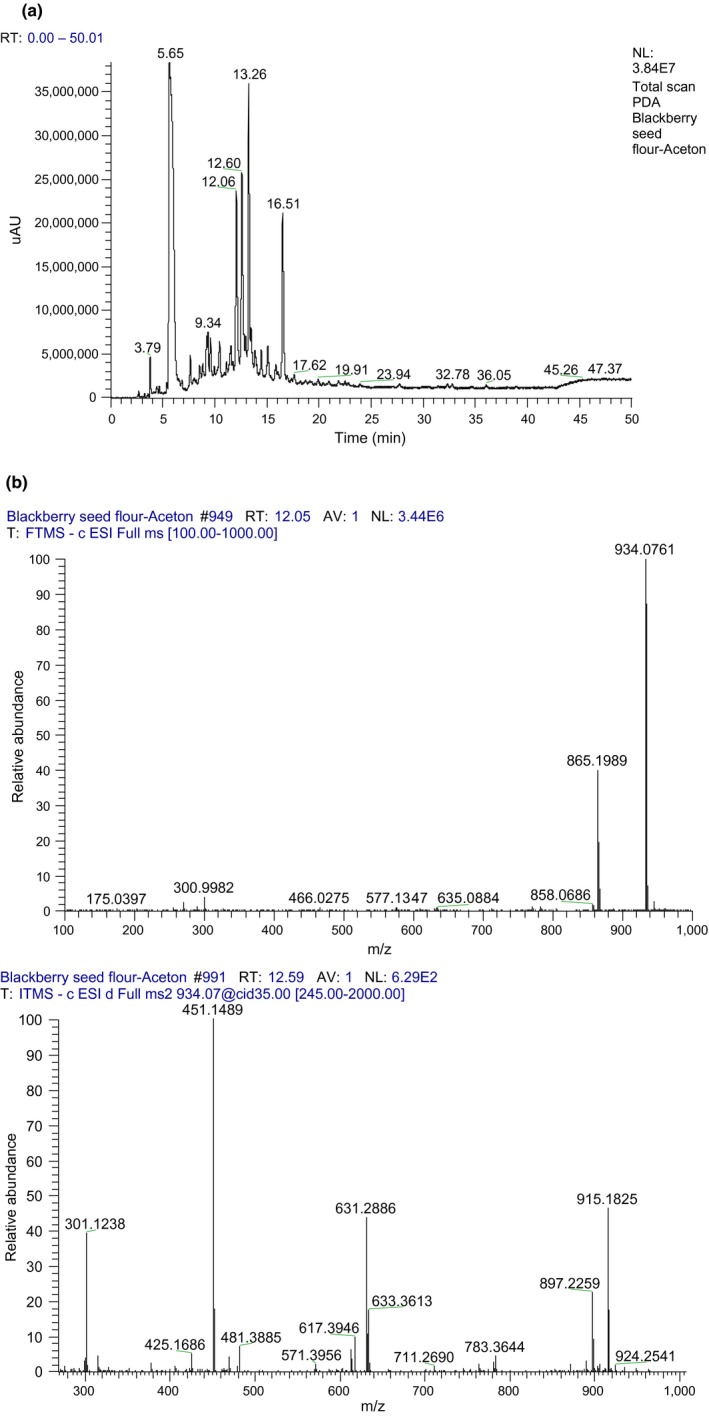
(a) Typical UHPLC chromatogram showing peaks of major compounds identified in the blackberry seed flour extract and their corresponding retention times; (b) MS and MS2 spectra of sanguiin H6 and peaks of fragmental ions in a negative mode

In the blackberry seed flour extract, thirteen compounds including hexahydroxydiphenic acid (HHDP) hexoside, pedunculagin isomers, galloyl‐HHDP‐hexoside, procyanidin B1, sanguiin H6 isomers, kaempferol‐3‐glucoside, ellagic acid, and ellagic acid derivatives were identified with sanguiin H6 as the primary compound (Table 1 and Figure 1). In addition to the chemical composition, this study quantified the selected compounds in the blackberry seed flour extract (Table 1). The primary compound sanguiin H6’s concentration was 457–675 μg ellagic acid equivalents/g (μg EAE/g). The second compound was ellagic acid, and concentration was 654 μg/g. The following compounds were ellagic acid derivatives and pedunculagin, at levels of 262–374 and 187 μg EAE/g, respectively (Table 1).

**Table 1:** Characterization of compounds present in blackberry seeds

Peak ID	*T*_R (min)_	Theor. [M‐H]^−^	Exptl. [M‐H]^ −^	Chemical formula	Tentative identification	Concentration (µg/g)
1	3.79	481.0618	481.0621	C_20_H_18_O_14_	Hexahydroxydiphenic acid (HHDP) hexoside	tr
2	7.64	783.0681	783.0687	C_34_H_24_O_22_	Pedunculagin isomer	186.65 ± 3.28 EAE
3	8.54	633.0728	633.0730	C_27_H_22_O_18_	Galloyl‐HHDP‐hexoside	tr
4	9.20	783.0681	783.0687	C_34_H_24_O_22_	Pedunculagin isomer	tr
5	9.23	783.0681	783.0694	C_34_H_24_O_22_	Pedunculagin isomer	tr
6	11.55	577.1346	577.1349	C_30_H_26_O_12_	Procyanidin B1	tr
7	12.06	1868.1425	934.0761*^a^*	C_82_H_54_O_52_	Sanguiin H6 isomer	457.03 ± 40.64 EAE
8	12.60	1868.1425	934.0692*^a^*	C_82_H_54_O_52_	Sanguiin H6 isomer	565.91 ± 48.91 EAE
9	13.26	1868.1425	934.0757*^a^*	C_82_H_54_O_52_	Sanguiin H6 isomer	675.10 ± 47.32 EAE
10	13.84	447.0927	447.0939	C_21_H_20_O_11_	Kaempferol−3‐glucoside	tr
11	14.44	433.0407	433.0418	C_19_H_14_O_12_	Ellagic acid pentoside isomer	373.57 ± 14.03 EAE
12	15.07	433.0407	433.0420	C_19_H_14_O_12_	Ellagic acid pentoside isomer	261.61 ± 9.83 EAE
13	16.51	300.9984	300.9983	C_14_H_6_O_8_	Ellagic acid	653.81 ± 66.84

Abbreviations: *T*_R (min)_, Retention Time; Theor. [M‐H]^‐^, Theoretical m/z of molecular ions; Exptl. [M‐H]^‐^, Experimental m/z of molecular ions; EAE, Ellagic acid equivalent; tr, Trace. *^a^*Experimental m/z values of [M‐2H]^‐2^.

The most abundant component, sanguiin H6, found in blackberry seed flour extract had a [M − H]^−^ of 1868.1425, which corresponds to the formula C_82_H_54_O_52_ (Table 1). A detailed analysis of the fragmental ions had peaks of *m/z* 783.3644 due to the loss of digalloyl‐gallagyl‐hexoside, *m/z* 633.3613 due to the loss of di‐HHDP‐glucose‐galloyl‐ellagic acid, and *m/z* 301.1238 due to the loss of galloyl‐glucose residue from galloyl‐HHDP‐hexoside in a negative mode (Figure 1b). This fragmentation matched with sanguiin H6 reported from a previous study (Mena et al., 2012). Therefore, this compound was tentatively identified as sanguiin H6. Correspondingly, the high resolution ESI‐MS of ellagic acid had an *m*/*z* of 300.9984 of [M − H]^−^ corresponding to the formula of C_14_H_6_O_8_ (Table 1). A detailed analysis of the fragmental ions of ellagic acid had peaks of 284.1061, 257.1262, 229.0817, and 185.1314 in a negative mode (Figure S1) (Mena et al., 2012). Based on ellagic acid standard's retention time and reported fragmental ions, this compound was tentatively determined as ellagic acid.

Two major compounds found in blackberry seed flour, pedunculagin and sanguiin H6, are ellagitannins. The ellagitannins are water soluble and high molecular weight phenolic compounds. When exposed to acids or bases, ester bonds are hydrolyzed and the hexahydroxydiphenic acid (HHDP) spontaneously rearranges into the water insoluble ellagic acid. Therefore, to quantify the ellagitannins found in blackberry seed flour extract, ellagic acid was used as the standard. In the current study, the total ellagic acid concentration after hydrolysis was about 3 mg/g of the dry seed flour (Table 1). Previously, it has been reported that ellagic acid concentration in blackberry after hydrolysis was 1.5 mg/g dry weight (Daniel et al., 1989). Interestingly, compared with the ellagic acid concentration found in the whole blackberry fruit, blackberry seed had a significantly greater ellagic acid concentration.

### 3.2 Potential effects of the blackberry seed flour extract on gut microbiota

**Figure 2:**
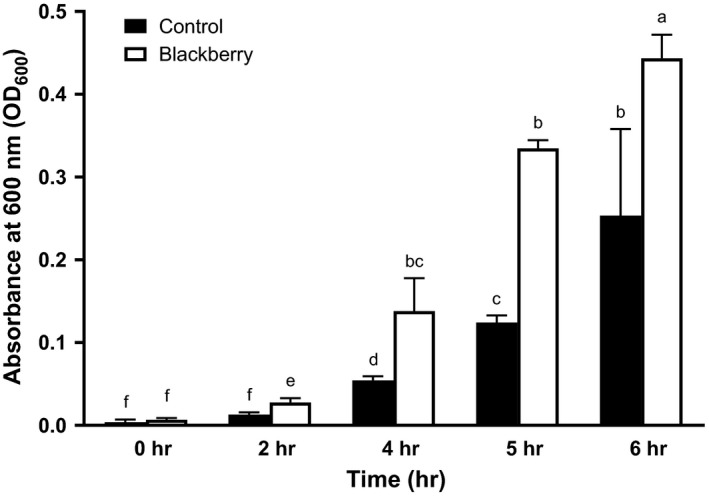
Effects of blackberry seed flour extract on gut bacterial growth. Each column represents the mean ± *SD* (*n* = 3). Columns marked with different letters are significantly different from each other at *p* ≤ .05

In this study, the blackberry seed flour extract was able to enhance gut bacterial growth in a time‐dependent manner (Figure 2). Previously, our group reported that extracts rich in polyphenols were able to enhance the gut bacterial populations (Choe et al., 2019). Similarly, Bialonska and others reported that the addition of pomegranate by‐product and pomegranate polyphenols significantly enhanced the growth of total bacteria (Bialonska et al., 2010). Also, it has been reported that several other polyphenols including punicalagin, punicalin, ellagic acid, gallic acid (Bialonska, Kasimsetty, Schrader, & Ferreira, 2009), catechin, epicatechin (Tzounis et al., 2007), and resveratrol (Larrosa et al., 2009) could interact with gut microbiota. In this study, the presence of pedunculagin, sanguiin H6, and ellagic acid in the blackberry seed flour extract (Table 1) might have close relation to the total bacterial growth since gut bacteria have been reported to utilizing these compounds (Bialonska et al., 2009).

**Figure 3:**
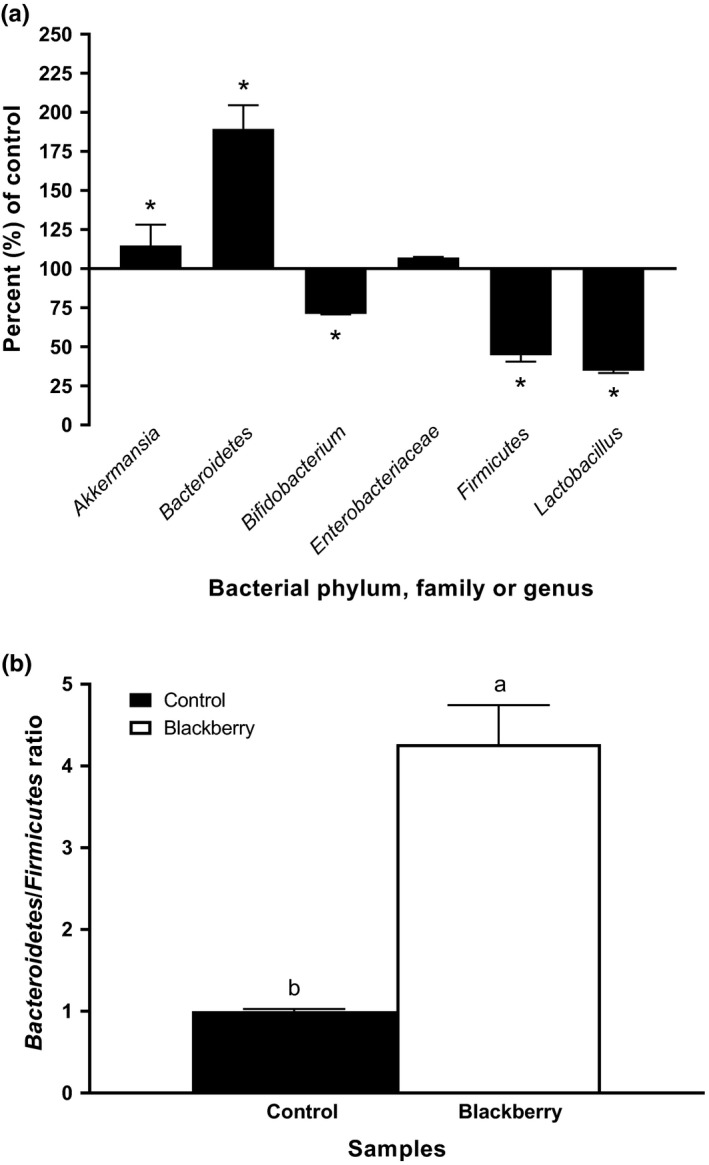
(a) Effects of the blackberry seed flour extract on the relative abundance of a specific bacterial phylum, family, or genus; (b) Effects of the blackberry seed flour extract on Bacteroidetes/Firmicutes ratio. Blackberry stands for blackberry seed flour extract. All values were normalized to the control. Each column represents the mean ± *SD* (*n* = 3). Columns marked with an asterisk are significantly different from the control at *p* ≤ .05. Columns marked with different letters are significantly different from each other at *p* ≤ .05

The blackberry seed flour extract was able to increase the abundance of *Bacteroidetes* phylum by two‐fold compared with the control (Figure 3a). In contrary, *Firmicutes* phylum was reduced by half compared with the control (Figure 3a). The *Bacteroidetes* and *Firmicutes* phyla are predominant phyla that consist of more than 90% of gut microbiota community in human. In addition, these two phyla are closely associated with human health. For example, it has been reported that *Bacteroidetes* phylum is known to carry very large number of genes that encode carbohydrate active enzymes (Flint, Scott, Duncan, Louis, & Forano, 2012). Also, *Bacteroidetes* phylum is involved in T‐cell activation, bile acid metabolism, and transformation of toxic and mutagenic compounds (Mazmanian, Round, & Kasper, 2008). The *Firmicutes* is associated with metabolism of fatty acid and aging (Ley et al., 2005). Ley and others found an increase in number of *Firmicutes* during aging progress (Ley et al., 2005). This increase in *Firmicutes* phylum alters the ratio between *Bacteroidetes* and *Firmicutes* phyla. In 2018, Spychala and others found that the ratio of *Firmicutes* to *Bacteroidetes* (F:B) increased about nine‐fold in aged mice compared with the young mice (Spychala et al., 2018). It is also recognized that the ratio of *Bacteroidetes* to *Firmicutes* phyla is associated with obesity. Ley and others found a significant increase of the *Firmicutes* and decrease of the *Bacteroidetes* levels in obese (ob/ob) mice compared with wild‐type mice (Ley et al., 2005). In the current study, the ratio of *Bacteroidetes* to *Firmicutes* increased over four‐fold in blackberry seed flour extract added culture compared with the control (Figure 3b). This result is consistent with the observation by Roopchand and others that grape polyphenols decreased the proportion of *Firmicutes* to *Bacteroidetes* in mice model (Roopchand et al., 2015). The result from this study proposes that blackberry seed flour may be used for body weight control and for health promotion in the aging population.

The blackberry seed flour extract also altered three bacterial genera and one family including *Akkermansia*, *Bifidobacterium*, *Lactobacillus*, and *Enterobacteriaceae*, respectively (Figure 3a). Among three genera, the blackberry seed flour extract increased *Akkermansia* genus (Figure 3a). *Akkermansia* genus is a mucin degrading bacterium that resides in the human intestinal tract and known as a contributor to the maintenance of gut health. Recent studies suggest that *Akkermansia* may reduce the risk of obesity, diabetes, and inflammation (Everard et al., 2013). Previously, it has been reported that grape polyphenols promote the growth of *Akkermansia* (Roopchand et al., 2015). Together, the results suggested the possible contribution of phenolics in the blackberry seed flour extract in increasing *Akkermansia* genus (Figure 3a). However, our previous studies found that polyphenols found in broccoli, carrot, cucumber, and milk thistle seeds decreased the abundance of *Akkermansia* genus (Choe et al., 2018, 2019), suggesting the specificity of the individual polyphenolic compound in interacting with a selected bacterial species.

The blackberry seed flour extract lowered the abundance of *Bifidobacterium* and *Lactobacillus* genera compared with the control (Figure 3a). *Bifidobacteria* and *Lactobacilli* are probiotic bacteria. *Bifidobacteria* are believed to benefit on host health. Due to their health‐beneficial properties, *Bifidobacteria* have been incorporated into many functional foods as active ingredients (O'Callaghan & Sinderen, 2016). *Lactobacilli* are well‐known probiotic bacteria. Previous studies suggest that together with *Bifidobacteria*, *Lactobacilli* can lower the concentration of carcinogenetic enzymes in colon flora through normalizing intestinal permeability and microflora balance as well as the production of antimutagenic organic acids and enhancing the host immune system (Kumar et al., 2010). The result from the current study is consistent with our previous studies that extracts rich in polyphenols decreased the abundance of both *Bifidobacterium* and *Lactobacillus* genera (Choe et al., 2018, 2019). Also, the result was supported by the observation of the previous study using grape seed extracts by Tabasco and others (Tabasco et al., 2011). Tabasco and others detected a growth inhibition effect of several lactic acid bacteria and *Bifidobacteria* using the grape seed extracts (Tabasco et al., 2011).

The blackberry seed flour extract did not change the abundance of *Enterobacteriaceae* family compared with the control (Figure 3a). The *Enterobacteriaceae* are a large family of gram‐negative bacteria, and some species naturally inhabit in human. Even though *Enterobacteriaceae* are nonspore‐forming bacteria, there are number of foodborne pathogens including *Salmonella*, *Yersinia enterocolitica*, pathogenic *Escherichia coli* (including *E. coli* O157:H7), *Shigella* spp., and *Cronobacter* spp. (Baylis, 2006). Thus, the increase of *Enterobacteriaceae* family in gut microbiota may cause health problems.

Recently, the gut microbiota has been spotlighted due to their health‐beneficial properties. Besides their health‐beneficial properties, the impact of gut microbiota on food metabolism has obtained significant attention. Ellagitannins found in fruits, legumes, and edible seeds are hydrolyzed to ellagic acid during digestion. Then, part of ellagic acid is metabolized by gut microbiota to produce urolithins (Landete, 2011). In 2017, Selma and others were able to isolate the bacterial species that is involved in ellagitannins and ellagic acid metabolism (Selma et al., 2017). Interestingly, it has been reported that urolithins, metabolites of ellagitannins and ellagic acid, showed anti‐inflammatory, anticarcinogenic, and free radical scavenging capacities (González‐Sarrías, Espín, Tomás‐Barberán, & García‐Conesa, 2009). These results suggest the benefits of consuming ellagitannins and ellagic acid rich foods including blackberry seed flours. To summarize, the current study showed that blackberry seed flour extract was able to change the gut microbiota profile and may have potential health benefits.

### 3.3 Free radical scavenging capacities

#### 3.3.1 Relative DPPH radical scavenging capacity (RDSC)

The blackberry seed flour extract demonstrated DPPH radical scavenging capacity with an RDSC value of 362 μmol TE/g (Table 2). This value is about three times greater than 137 μmol TE/g reported by Zafra‐Rojas and others for the blackberry residues consisted of peels, seeds, and pulp (Zafra‐Rojas et al., 2016). Also, the RDSC value obtained from the current study is compatible with the RDSC values of berry seeds that Parry and others previously reported (Parry et al., 2006). Parry and others tested black raspberry, red raspberry, blueberry, and cranberry seeds using 50% acetone as a solvent and found their RDSC values of 200, 510, 670, and 1,260 μmol TE/g, respectively (Parry et al., 2006). Blackberry seed flour had a DPPH scavenging capacity comparable to other berry seeds.

**Table 2:** Free radical scavenging capacities of cold‐pressed blackberry seed flour extract

	RDSC	ORAC	HOSC	ABTS
	(μmol TE/g)			
Blackberry	362.09 ± 22.07	304.01 ± 15.65	2,531.44 ± 375.09	266.82 ± 16.52

Abbreviations: Blackberry, blackberry seed flour extract; RDSC, relative DPPH scavenging capacity; ORAC, oxygen radical absorbing capacity; HOSC, hydroxyl radical scavenging capacity; ABTS, ABTS radical scavenging capacity; and TE, Trolox equivalent.

#### 3.3.2 Oxygen radical absorbing capacity (ORAC)

The blackberry seed flour extract showed an oxygen radical absorbing capacity of 304 μmol TE/g (Table 2). Previously, it has been reported that extracts of fruit seeds such as black raspberry, red raspberry, blueberry, and cranberry had ORAC values of 296, 276, 153, and 111 μmol TE/g, respectively (Parry et al., 2006). Compared with other berry seed flour extracts, blackberry seed flour extract showed high oxygen radical absorbing capacity.

#### 3.3.3 Hydroxyl radical (HO^•^) scavenging capacity (HOSC)

The blackberry seed flour extract showed HOSC value of 2,531 μmol TE/g (Table 2). This is the first HOSC report of blackberry seed flour. In 2017, Gao and others reported HOSC values of blueberry in a range of 566–1048 μmol TE/g (Gao et al., 2017). Compared with the whole blueberry fruit extract, blackberry seed flour extract showed a HOSC value 2.5‐ to 4.5‐fold greater. Previously, our group tested HOSC of vegetable seed flours such as broccoli, carrot, and cucumber using 50% acetone as a solvent (Choe et al., 2018). The broccoli, carrot, and cucumber seed flour extracts had HOSC values of 270, 112, and 52 μmol TE/g, respectively (Choe et al., 2018). Compared with vegetable seed flours, the blackberry seed flour had a significantly greater HOSC value.

#### 3.3.4 ABTS^•+^ scavenging capacity

The blackberry seed flour extract showed an ABTS^•+^ scavenging capacity value of 267 μmol TE/g (Table 2). This value is compatible with the black raspberry seed extract previously reported (Parry & Yu, 2006). In that study, Parry and Yu used two different solvents, 100% ethanol and 50% acetone, for the extraction and found ABTS^•+^ scavenging capacity values of 233 and 361 μmol TE/g, respectively (Parry & Yu, 2006).

Free radicals are generated inside the human body internally and externally. Internal sources may include mitochondria, xanthine oxidase, peroxisomes, inflammation, phagocytosis, and exercise. External sources include smoking, environmental pollutants, radiation, certain drugs, industrial solvents, and ozone. Either from internal or external, excessive free radicals in the human body can cause oxidative stress, a deleterious process which can vigorously change the cell membranes, lipids, lipoproteins, and DNA. Thus, scavenging free radicals using dietary intervention is regarded as a potential health benefit. In this study, blackberry seed flour extract showed promising free radical scavenging capacities. This result suggests that blackberry seed flour may be used for diminishing the risk of oxidative stress‐caused human chronic diseases.

### 3.4 Anti‐inflammatory capacity

**Figure 4:**
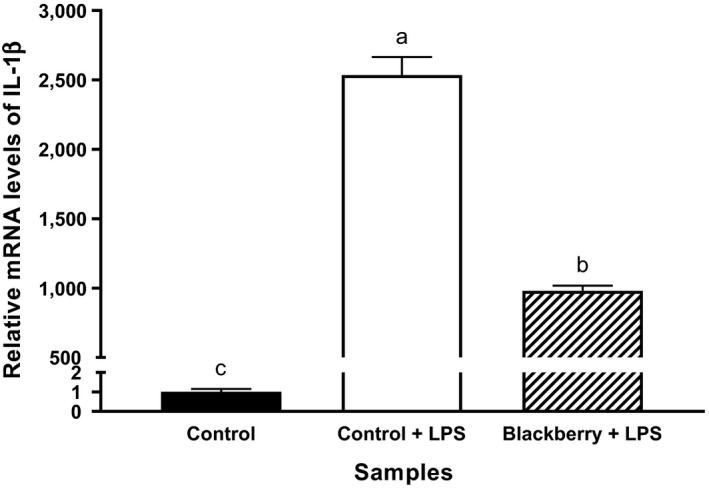
An anti‐inflammatory capacity of the blackberry seed flour extract in J774A.1 mouse macrophage cells: interleukin 1 beta (IL‐1β). Blackberry stands for blackberry seed flour extract. All values were normalized to the control. Each column represents the mean ± *SD* (*n* = 3). Columns marked with different letters indicate a significant difference at *p* ≤ .05

In the current study, blackberry seed flour extract showed significant inhibition of pro‐inflammatory marker gene, interleukin 1 beta (IL‐1β), induced by lipopolysaccharides (LPS) compared with the LPS stimulated control J774A.1 mouse macrophages (Figure 4). Growing evidence suggests that chronic health conditions such as diabetes, cardiovascular disease, cancer, rheumatoid arthritis, and inflammatory bowel disease are linked to inflammation (Clark, Kroger, & Tisch, 2017). Therefore, inflammation has been considered a target for reducing the risk of chronic diseases. During the process of inflammation, IL‐1β plays an important role. IL‐1β is a potent pro‐inflammatory cytokine and a key mediator of the inflammatory response. Previous studies reported that blocking or inhibiting IL‐1β can significantly lower the risk of chronic diseases (Dinarello, Simon, Jos, & van der Meer, 2012). Also, ellagic acid, a compound identified in blackberry seed flour extract (Table 1), has been reported for its anti‐inflammatory capacity (Chen, Chen, & Zhou, 2018; El‐Shitany, El‐Bastawissy, & El‐desoky, 2014). The mechanism of action for ellagic acid's anti‐inflammatory capacity is still not clear. But, it is believed that ellagic acid modulates the production of cyclooxygenase‐2 (COX‐2) mRNA mainly through the inhibition of reactive oxygen species (ROS) production which in turn inhibited nuclear kappa light‐chain enhancer of activated B cells (NF‐ κB) activation (El‐Shitany et al., 2014). The other possible mechanism of action is by blocking the COX‐2 receptor. El‐Shitany and others tested molecular docking between ellagic acid and COX‐2 active site and found high affinity. The binding affinity of ellagic acid and COX‐2 was even higher than that of anti‐inflammatory drugs including diclofenac and meloxicam (El‐Shitany et al., 2014). Taking together, components found in blackberry seed flour may abate the risk of IL‐1β mediated inflammation and related chronic diseases.

### 3.5 Antiproliferative capacity

The blackberry seed flour extract at an initial treatment concentration of 0.4 mg flour equivalents/mL suppressed the growth of LNCaP prostate cancer cells after 48 hr (Figure S2). Previously, it has been reported that ellagic acid treatment induced a marked increase in DNA damage in LNCaP and DU145 prostate cancer cell lines in a dose‐dependent manner (Vanella, Barbagallo, et al., 2013a). The mechanism of action of ellagic acid's antiproliferative capacity includes several signaling pathways. In cancer cells, aberrant accumulation of intracellular β‐catenin is a well‐recognized characteristic. Vanella and others found a reduction in β‐catenin levels when LNCaP prostate cancer cells were treated with ellagic acid. Also, they found that ellagic acid was able to reduce the mammalian target of rapamycin (mTOR) activation. Previously, the capacity of phospho‐protein kinase B (p‐Akt) to phosphorylate/activate mTOR has been described in several cancer cell lines (Diersch et al., 2013; Pratheeshkumar et al., 2012). Their finding suggests that ellagic acid exerts an antiproliferative effect by reducing PI3K/Akt downstream signaling through inhibition of mTOR phosphorylation (Vanella, Giacomo, et al., 2013b). The second possible mechanism involves NAD‐dependent deacetylase sirtuin‐1 (SIRT1) protein. SIRT1 functions as an oncogenic protein and plays a role in tumorigenesis (Chen, Jeng, Yuan, Hsu, & Chen, 2012). This protein is overexpressed in human prostate cancer cells including DU145, LNCaP, 22Rv1, and PC3. In the LNCaP cell, the treatment of 25 and 50 μM of ellagic acid significantly reduced the SIRT1 protein level (Vanella, Giacomo, et al., 2013b).

Ellagitannins and ellagic acid also had antiproliferative capacities in liver, skin, esophageal and oral, cervical, lung, breast, and colon cancer cell lines (Ismail et al., 2016). These results suggest the potential use of blackberry seed flour in reducing the risk of carcinogenesis.

## 4 CONCLUSIONS

The current study identified the chemical composition of blackberry seed flour and evaluated its potential health‐beneficial properties including gut microbiota modulatory, free radical scavenging, anti‐inflammatory, and antiproliferative capacities. The presence of ellagitannins and ellagic acid in blackberry seed flour and their potential health‐beneficial properties suggest the potential value‐added utilization of blackberry seed flour in enhancing human health.

## CONFLICT OF INTEREST

None declared.

## ETHICAL APPROVAL

This study does not involve any human or animal testing.
