# Supplementary material for: Chemical composition of cold‐pressed blackberry seed flour extract and its potential health‐beneficial properties
Source: Food Sci Nutr. 2020 Jan 20;8(2):1215–25. doi: 10.1002/fsn3.1410 (PMC7020322; doi:10.1002/fsn3.1410)
Supplement: Supplementary file 1 [file FSN3-8-1215-s001.docx]

**Chemical composition of cold-pressed blackberry seed flour extract, and its potential health beneficial properties**

**Authors and affiliations:**

Uyory CHOE^1,2^, [uchoe@umd.edu](mailto:uchoe@umd.edu)
Yanfang LI^3,4,5^, [yanfangli0820@163.com](mailto:yanfangli0820@163.com)

Lu YU^1,2^, [yulu0640514@gmail.com](mailto:yulu0640514@gmail.com)

Boyan GAO^*4^, [gaoboyan@sjtu.edu.cn](mailto:gaoboyan@sjtu.edu.cn)

Thomas T. Y. WANG^2^, [Tom.Wang@ars.usda.gov](mailto:Tom.Wang@ars.usda.gov)

Jianghao SUN^3^, [Jianghao.Sun@ars.usda.gov](mailto:Jianghao.Sun@ars.usda.gov)

Pei CHEN^3^, [Pei.Chen@ars.usda.gov](mailto:Pei.Chen@ars.usda.gov)

Liangli YU^1^, [lyu5@umd.edu](mailto:lyu5@umd.edu)

^1^Department of Nutrition and Food Science, University of Maryland, College Park, MD 20742, USA

^2^Diet, Genomics and Immunology Laboratory, Beltsville Human Nutrition Research Center, Agricultural Research Service, United States Department of Agriculture, Beltsville, MD 20705, USA

^3^Food Composition and Methods Development Laboratory, Beltsville Human Nutrition Research Center, Agricultural Research Service, United States Department of Agriculture, Beltsville, MD 20705, USA
^4^Institute of Food and Nutraceutical Science, School of Agriculture and Biology, Shanghai Jiao Tong University, Shanghai 200240, China
^5^Beijing Advanced Innovation Center for Food Nutrition and Human Health, Beijing Technology & Business University (BTBU), Beijing 100048, China

*Correspondence: Liangli Yu, Department of Nutrition and Food Science, University of Maryland, College Park, MD 20742, USA; Boyan Gao, Institute of Food and Nutraceutical Science, School of Agriculture and Biology, Shanghai Jiao Tong University, Shanghai 200240, China.
Emails: lyu5@umd.edu;
gaoboyan@sjtu.edu.cn.

**FIGURE CAPTIONS**

**Figure S1** MS and MS^2^ spectra of ellagic acid and peaks of fragmental ions in a negative mode.

**Figure S2** Anti-proliferative capacity of the blackberry seed flour extract in LNCaP prostate cancer cells. A final concentration of 0.4 mg flour equivalent/mL blackberry seed flour extract was treated in LNCaP prostate cancer cells. LNCaP prostate cancer cell numbers were measured every 24 h. Each column represents the mean ± SD (n = 3). Columns marked with different letters indicate significant difference at *p* ≤ .05.

**Figure S1**

(A)

(B)

**Figure S2**
